# Complete Heart Block in a Patient Undergoing Combination Immune Checkpoint Inhibitor Therapy

**DOI:** 10.7759/cureus.66776

**Published:** 2024-08-13

**Authors:** Himara Koelmeyer, Kinley Buckley, Denise Feradov, Nicholas Kotch

**Affiliations:** 1 Internal Medicine, University of South Florida Morsani College of Medicine, Tampa, USA; 2 Cardiovascular Medicine, University of South Florida Morsani College of Medicine, Tampa, USA; 3 Electrophysiology, University of South Florida Morsani College of Medicine, Tampa, USA

**Keywords:** cardio-oncology, chemotherapy-related toxicity, immune-checkpoint inhibitor (ici), myocarditis, complete heart block

## Abstract

Combination immune checkpoint inhibitor (ICI) therapy is an emerging chemotherapy strategy for patients with solid tumor malignancies. Cardiotoxicity is a rare adverse effect of ICI therapy, most commonly presenting as acute myocarditis and, less frequently, as significant conduction abnormalities. We present a unique case of a 68-year-old female with urothelial cancer who developed shortness of breath and chest pain one week after receiving combination ICI therapy with ipilimumab and nivolumab. Biomarkers were elevated, including high-sensitivity troponin to 14,000 ng/L and creatine phosphokinase to 20,000 U/L. Due to suspicion of acute ICI-related myocarditis, a transthoracic echocardiogram (TTE) was obtained and demonstrated preserved ejection fraction (EF). Pulse-dose methylprednisolone therapy was initiated. However, the patient's clinical status continued to decline, and she developed bradycardia due to a complete heart block (CHB). This was initially treated with a dopamine infusion, but due to hypotension and hemodynamic instability, a transvenous pacemaker was placed. She continued to decline from a heart failure standpoint and developed acute hypoxic respiratory failure, requiring intubation due to pulmonary edema. A repeat TTE acquired three days following the initial echocardiogram demonstrated a newly reduced EF of 30%-35%. Additional anti-inflammatory agents were administered, including mycophenolate, infliximab, and anti-thymocyte globulin, with little improvement in clinical status. Unfortunately, she rapidly deteriorated, resulting in pulseless electrical activity (PEA) arrest and circulatory death. The autopsy revealed severe biventricular myocarditis with partial involvement of the atrioventricular node, consistent with her clinical syndrome of acute heart failure and CHB. A literature review demonstrated very few cases of ICI-related CHB. This case highlights a rare instance of atrioventricular dissociation in a patient with cardiotoxicity due to combination ICI therapy.

## Introduction

Immune checkpoint inhibitor (ICI) therapy is used to treat several malignancies, including melanoma, renal cell carcinoma, non-small cell lung cancer, Hodgkin's lymphoma, head and neck cancers, gastrointestinal malignancies, genitourinary malignancies, and hepatocellular carcinoma [1]. There are seven ICIs approved for use by the United States Food and Drug Administration (FDA), including ipilimumab, nivolumab, pembrolizumab, cemiplimab, nivolumab, atezolizumab, and durvalumab [2]. Mechanistically, ICIs inhibit checkpoints that suppress the immune response, thereby enhancing the immune system's ability to destroy cancer cells. The specific checkpoints targeted by ICIs are cytotoxic T-lymphocyte antigen-4 (CTLA-4), programmed death-1 (PD-1), and programmed death-ligand 1 (PD-L1). Combination ICI therapy inhibits two checkpoints, rather than one, to further augment the immune response for targeted antitumor therapy. ICI-related cardiotoxicity is not a common adverse effect, with an incidence of 0.04% to 1.14%, but it has a high mortality rate of 25%-50% [2]. The risk factors for cardiotoxicity are not well understood; however, the risk is higher with combined therapy; some studies have shown an almost doubled rate of mortality [2-4]. Other risk factors possibly associated with an increased risk of cardiovascular events include female sex, African American race, and tobacco use [5]. Cardiotoxicity most commonly presents as myocarditis, whereas severe conduction abnormalities such as complete heart block (CHB) have historically been rare. Emerging data suggest that ICI myocarditis can present with new conduction blocks. This case describes a patient undergoing combination ICI therapy who developed myocarditis and complete atrioventricular dissociation.

## Case presentation

A 68-year-old female with a past medical history of stage IV urothelial cancer, coronary artery disease with a prior stent to the left anterior descending artery (LAD), alcoholic cirrhosis, chronic stage 3b kidney disease secondary to hypertensive nephrosclerosis and hepatorenal syndrome, hypertension, and non-insulin-dependent type 2 diabetes presented to an outside facility due to progressive shortness of breath and chest discomfort one week after receiving cycle one of combination ICI therapy with ipilimumab and nivolumab, in addition to sacituzumab govitecan. Vital signs were significant for a blood pressure of 220/112 mmHg, requiring a nitroglycerin infusion for the treatment of hypertensive urgency, and a heart rate of 112 beats per minute (bpm). She was found to have significantly elevated biomarkers, including high-sensitivity troponin (hs-Tn) of 14,000 ng/L (reference range: <14 ng/L) and creatine phosphokinase (CPK) of 20,000 U/L (reference range: 29-168 U/L). Other significant lab results included creatinine 1.59 mg/dL (reference range: 0.57-1.11 mg/dL), aspartate aminotransferase (AST) 983 U/L (reference range: 15-37 U/L), and alanine aminotransferase (ALT) 401 U/L (reference range: 13-56 U/L) (Table 1).

**Table 1:** Laboratory findings

Hospital Day	1	2	3	4	6	7	8	9	Reference Range
High-sensitivity troponin (hs-Tn)	14,146	14,610	>25,000	>25,000	43,992	27,904	19,891	22,701	0-59 ng/L
B-type natriuretic peptide (BNP)	-	-	-	-	-	-	1,315	-	<100 pg/mL
Creatine phosphokinase (CPK)	26,793	30,211	17,834	4,689	-	3,650	4,002	4,102	26-192 U/L
Aspartate aminotransferase (AST)	811	1,054	983	791	520	-	1,430	2,677	15-37 U/L
Alanine aminotransferase (ALT)	221	343	402	397	322	-	1,453	2,063	13-56 U/L
Creatinine	1.52	1.59	1.93	2.81	3.2	4.2	1.4	0.8	0.55-1.02mg/dL

**Figure 1:**
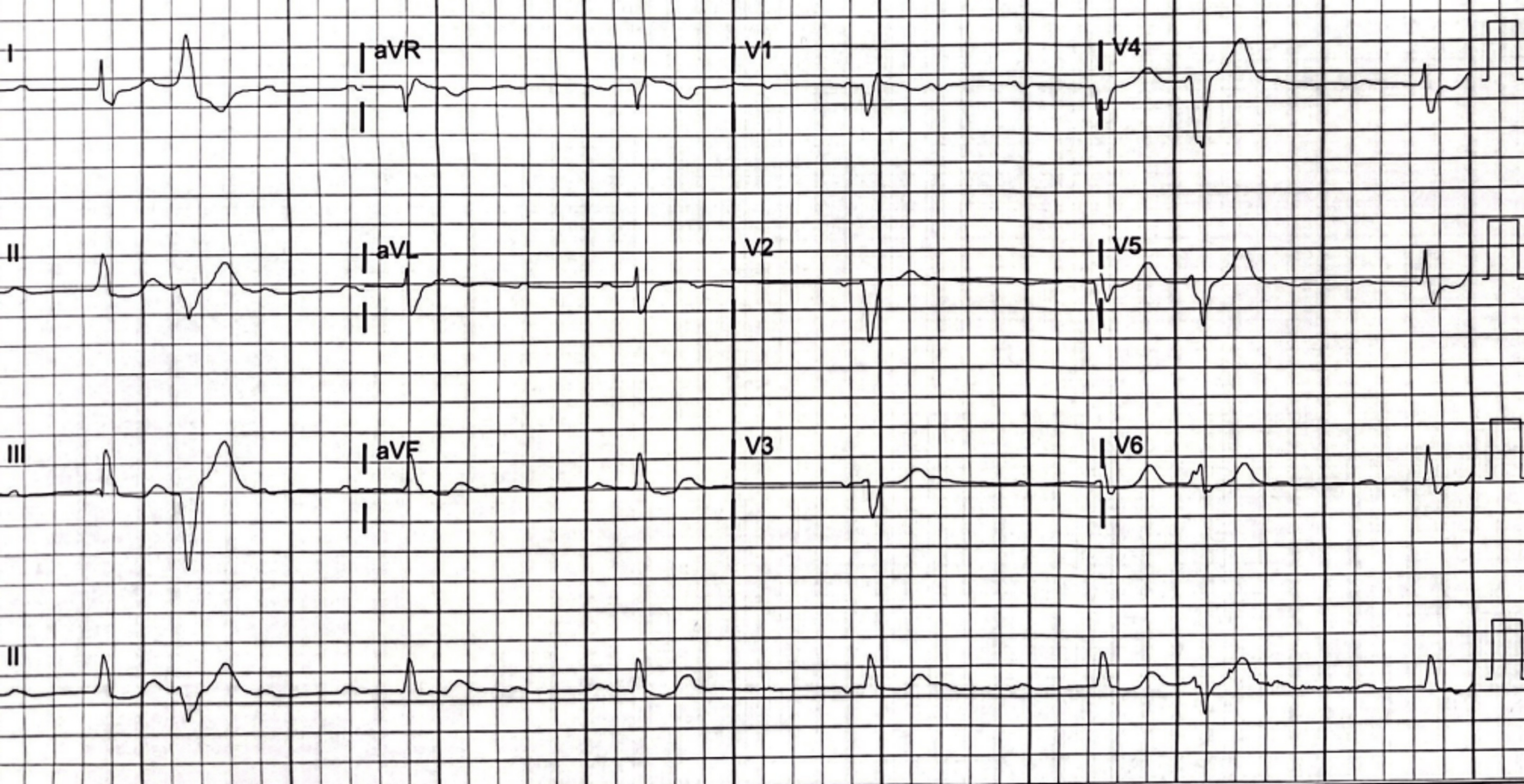
Complete heart block

An electrocardiogram (EKG) showed sinus tachycardia and no acute ischemic changes. A transthoracic echocardiogram (TTE) was obtained on hospital day two and revealed an ejection fraction (EF) of 60%-65% and no regional wall motion abnormalities. A left heart catheterization was performed given elevated troponin and chest discomfort, which showed no obstructive coronary disease and a patent LAD stent. Given the elevated biomarkers and clinical symptoms of heart failure, there was high suspicion of ICI-related myocarditis, and high-dose methylprednisolone was administered. Despite therapy, hs-Tn continued to increase to 25,000 ng/L, creatinine further rose to 2.8 mg/dL, while AST/ALT increased to 791/397 U/L (Table 1). Due to the lack of improvement in troponin and multiorgan failure, mycophenolate was administered in addition to high-dose methylprednisolone as an additional anti-inflammatory agent. She then developed CHB (Figure 1) on hospital day four, initially asymptomatic and hemodynamically stable. However, the patient became hypotensive with a blood pressure of 85/43 mmHg (mean arterial pressure (MAP) of 57 mmHg), bradycardic at 32 bpm, and somnolent. This prompted a dopamine infusion initiation, resulting in improved blood pressure (MAP >65 mmHg) and clinical status. On hospital day five, she became hypotensive, bradycardic, and somnolent despite dopamine infusion, requiring the placement of a transvenous pacemaker (TVP). The patient was then transferred to our tertiary care center facility for escalation of care.

**Figure 2:**
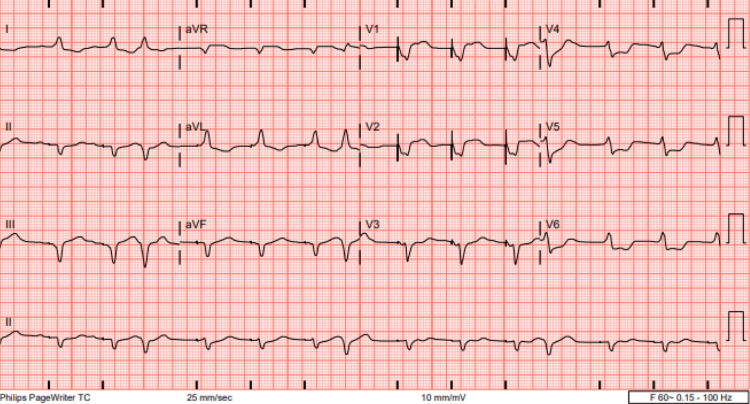
Ventricular-paced rhythm

On arrival at our facility on hospital day 6, she was hemodynamically stable with a blood pressure of 133/77 mmHg and a heart rate set to 70 bpm on TVP (Figure 2), with SpO_2_ >95% on room air. At this time, CPK had risen to 3,777 U/L (reference range: 29-168 U/L), while hs-Tn had further increased to 43,992 ng/L (reference range: 0-59 ng/L), and B-type natriuretic peptide (BNP) was measured at 1,315 pg/mL (reference range: <100 pg/mL).

Labs demonstrated worsening renal function (creatinine 3.2 mg/dL); however, AST/ALT showed continued improvement (520/322 U/L) (Table 1). A repeat TTE was obtained on hospital day six and demonstrated a decline in EF to 30%-35% (Video 1).

**Video 1:** Echocardiogram demonstrating a reduced ejection fraction

She subsequently decompensated further from a heart failure standpoint and developed significant pulmonary edema and acute hypoxic respiratory failure requiring endotracheal intubation. Due to worsening renal function and hyperkalemia, renal replacement therapy was also initiated. Infliximab was added as a third anti-inflammatory agent. A right heart catheterization was performed, which demonstrated elevated intracardiac filling pressures but a compensated cardiac index and output. An endomyocardial biopsy was obtained, which revealed active lymphocytic myocarditis with myocyte necrosis. Due to the worsening clinical status, the new vasopressor requirement (norepinephrine infusion), and the persistently elevated hs-Tn levels (22,701 ng/L), antithymocyte globulin (ATG) was administered as a fourth and final anti-inflammatory agent. Unfortunately, the patient's condition declined rapidly despite multiple lines of therapy, resulting in pulseless electrical activity (PEA) arrest and circulatory death on hospital day nine.

## Discussion

To our knowledge, there are only 14 reported cases of CHB due to ICI therapy, the earliest being in 2018. In most cases, patients were treated with ICI monotherapy, while three received combination therapy. Of those receiving monotherapy, the majority improved with high-dose glucocorticoids. Clinical outcomes and mortality were worse in those receiving combination therapy; three of four patients died, as did our patient [6-8]. This is reflected in the literature, which differentiates mortality by combination versus ICI monotherapy, demonstrating a mortality rate of 65.6% versus 44.4%, respectively [4]. Another retrospective study revealed a threefold higher risk of myocarditis in those receiving combination therapy, with an incidence of 0.27% compared to 0.09% in those receiving monotherapy [5].

Myocarditis is a rare complication of ICI therapy, with an incidence of 0.04%-1.14% and often occurring within 30 days of the first or second cycle of therapy [2,4], carrying a mortality rate as high as 50% [2,9]. It can present with various symptoms, including asymptomatically elevated biomarkers that represent myocardial injury (e.g., troponin, creatine kinase), chest pain, acute decompensated heart failure, and cardiogenic shock [9,10]. In a review by Mahmood et al., it was found that among those presenting with myocarditis, 94% had elevated troponin, 89% had an abnormal EKG, and 51% had a preserved EF (>50%) [11]. The degree of troponin elevation has been found to be a reasonable predictor of morbidity in these patients [2]. One retrospective multicenter study found that patients with ICI myocarditis who developed CHB were at a higher risk of all-cause mortality at 30 days than those who did not (48% vs. 22.1%, respectively) [12].

The mechanism of ICI myocarditis is unclear, but hypotheses include shared antigens between the targeted malignancy and myocardium, as well as T cells targeting similar or dissimilar muscle antigens [2]. Another proposed mechanism is increased immune-mediated activity, which allows for an exaggerated T-cell response and antigen recognition in non-target tissues, increasing circulating cytokines and the formation of autoantibodies in non-target tissues [4,9,11], leading to subsequent tissue inflammation. This was reflected in our patient, whose endomyocardial biopsy pathology revealed infiltration of T lymphocytes and macrophages. Similarly, in two other cases, pathology revealed T-cell infiltration [6,7]. Conduction system involvement is rare but is likely a result of myocarditis extending from the muscle to the electrical system. This was confirmed in our patient, whose autopsy revealed severe, extensive myocarditis in the bilateral ventricles and interventricular septum with partial involvement of the atrioventricular (AV) node, explaining her clinical syndrome of acute decompensated heart failure and CHB. In some cases, patients presented with sole conduction abnormalities without heart failure [13-16], with 75% improving with high-dose corticosteroid therapy.

Management of ICI myocarditis includes high-dose corticosteroid therapy, typically with methylprednisolone. However, in the setting of clinical deterioration, additional anti-inflammatory agents may be utilized. Given the predominantly T-cell-mediated inflammation, agents such as tacrolimus and ATG have also been utilized in refractory cases [9,17]. Tacrolimus predominantly suppresses T cells by inhibiting calcineurin, a key factor in T-cell activation. ATG is an immunosuppressive agent derived from the serum of rabbits or horses; in our patient's case, rabbit ATG was used. In this process, rabbits or horses are immunized with human thymocytes (immature T cells in the thymus), leading to the formation of antibodies to these thymocytes that can be isolated to create ATG [18]. This agent is particularly useful in ICI myocarditis given its ability to target T-cell-mediated inflammation, which aligns with our patient's autopsy findings of extensive myocarditis with T-cell infiltration. Additionally, as demonstrated in our patient, mycophenolate mofetil (MMF) is used in refractory cases as an adjunct to high-dose corticosteroids. MMF works by impairing DNA and RNA synthesis, which inhibits the proliferation of B and T cells, thereby suppressing the immune response and providing significant anti-inflammatory effects [19]. Although MMF has a slower onset of action, its use in conjunction with high-dose corticosteroids allows for rapid control of acute inflammation and long-term immune suppression, addressing both short- and long-term anti-inflammatory needs. Our patient likely did not improve despite multiple therapies due to significant and rapidly progressive myocardial involvement, as noted in the autopsy. Thus, early recognition may be vital in mitigating the high rates of mortality in ICI-related myocarditis.

Given the high mortality of ICI-related myocarditis, further research is imperative to understand which patients are at the highest risk, as this knowledge could guide therapy selection, closer monitoring, and early intervention if myocarditis is suspected. Combination therapy is a significant risk factor for more severe disease and higher mortality [4], with additional risk factors including female gender, African American race, and tobacco use [5]. Given the variety in clinical presentation and disease severity, it is important to maintain a high degree of clinical suspicion in patients receiving these therapies.

## Conclusions

Fulminant myocarditis is a well-described complication of ICI therapy in the literature. However, CHB is a less frequently documented adverse outcome related to cardiotoxicity. Our case involves a patient with urothelial carcinoma who was treated with combination ICI therapy using ipilimumab and nivolumab. This case highlights the well-known risk factor of severe myocarditis associated with ICI therapy, given the combined ICI approach and subsequent severe cardiac manifestations, including acute decompensated heart failure and CHB. Management involves cessation of ICI therapy, high-dose corticosteroids, and potentially additional immunosuppressive agents. TVP placement is indicated in cases of CHB in patients who do not improve with anti-inflammatory therapy and are hemodynamically unstable. Given the various clinical presentations and high mortality rate of ICI-related myocarditis, especially in those with concomitant CHB, it is imperative to maintain a high index of clinical suspicion in at-risk patients to facilitate early diagnosis and prompt treatment.
